# Unsteady MHD Thin Film Flow of an Oldroyd-B Fluid over an Oscillating Inclined Belt

**DOI:** 10.1371/journal.pone.0126698

**Published:** 2015-07-06

**Authors:** Taza Gul, Saeed Islam, Rehan Ali Shah, Asma Khalid, Ilyas Khan, Sharidan Shafie

**Affiliations:** 1 Mathematics Department, Abdul Wali Khan University, Mardan, KPK Pakistan; 2 Mathematics Department, University Engineering Technology (UET), Peshawar, KPK Pakistan; 3 Basic Sciences Department, College of Engineering, Majmaah University, P.O. Box 66, Majmaah, 11952, Saudi Arabia; 4 Department of Mathematical Sciences, Faculty of Science, Universiti Teknologi Malaysia, Skudai Johor, 81310, Malaysia; Massachusetts Institute Of Technology, UNITED STATES

## Abstract

This paper studies the unsteady magnetohydrodynamics (MHD) thin film flow of an incompressible Oldroyd-B fluid over an oscillating inclined belt making a certain angle with the horizontal. The problem is modeled in terms of non-linear partial differential equations with some physical initial and boundary conditions. This problem is solved for the exact analytic solutions using two efficient techniques namely the Optimal Homotopy Asymptotic Method (OHAM) and Homotopy Perturbation Method (HPM). Both of these solutions are presented graphically and compared. This comparison is also shown in tabular form. An excellent agreement is observed. The effects of various physical parameters on velocity have also been studied graphically.

## Introduction

In recent time, non-Newtonian fluids have become quite prevalent in industry and engineering. Some of their common examples are polymer solutions, paints, certain oils, exocitic lubricants, colloidal and suspension solutions, clay coatings and cosmetic products. As a consequence of diverse physical structures of these fluids, there is not even a single constitutive model which can predict all the salient features of non-Newtonian fluids. Generally there are three non-Newtonian fluids models. They are known as (i) the differential type, (ii) the rate type, and (iii) the integral type. But the most famous amongst them are the first two models. In this work, we will study the second model, the rate type fluid and consider its subclass known as Oldroyd-B fluid. The simplest subclass of rate type fluid is Maxwell fluid, however, this fluid model can only be described in terms of its relaxation time, while there are no information on its retardation time. The Oldroyd-B fluid model, on the other hand, has a measurable retardation time and can relate the viscoelastic manners of dilute polymeric solutions under general flow conditions. Fetecau et al. [1] obtained exact solutions in their study on constantly accelerating flow over a flat plate for Oldroyd-B fluid. In the following year, Fetecau et al. [2] studied the transient oscillating motion of an Oldroyd-B fluids in cylindrical domains and obtained the exact solutions. Haitao and Mingyu [3] studied the series solution for the plane Poiseuille flow and plane couette flow of an Oldroyd-B fluid using the sine and Laplace transformations. Hayat et al. [4] investigated the exact solution of Oldroyd-B fluid for five different problems. Liu et al. [5] discussed the MHD flow of an Oldroyd-B fluid between two oscillating cylinders. Khan et al. [6, 7] investigated the solution for unsteady MHD flow of an Oldroyd-B fluid passing through a porous medium. They obtained the exact solutions for both of their problems by using the Laplace transform technique and discussed the physical behavior of relaxation and retardation times of fluid motion.

Burdujan [8] studied the unsteady flow of incompressible Oldroyd-B fluid between two cylinders. He obtained the exact solution by using Hankal and Laplace transformations. Asia et al. [9] investigated the oscillating motion of Oldroyd-B fluid between two sides wall. They obtained the starting solution of velocity field. Shahid et al. [10] examined the steady and unsteady flow of Oldroyd-B fluid. Steady state and transient solution have been obtained by using Laplace and Fourier series. Aksel et al. [11] discussed the flow of an Oldroyd-B fluid due to the oscillation of a plate. As a special case, they reduced their solutions to those of Maxwell and Newtonian fluids. Ghosh and Sana [12] analyzed hydromagnetic flow of an Oldroyd-B fluid near a pulsating plate. In subsequent papers, Ghosh and Sana [13] and Gosh et al. [14] discussed the unsteady flow of electrically conducting Oldroyd-B fluid induced by rectified sine pulses and half rectified sine pulses. Khan and Zeeshan [15] extended the work of Gosh and Sana [12] by taking the Oldroyd-B fluid into a porous medium. As discussed, most of Oldroyd-B fluid studies are confined to some specific geometries. Studies on Oldroyd-B fluids over an oscillating belt are scarce, especially when considering the thin film flow of an Oldroyd-B fluid over an inclined oscillating belt.

Having such motivation in mind, Gul et al. [16–18] studied the analytical solution of MHD thin film flow of non-Newtonian fluid on a vertical oscillating belt by using the ADM and OHAM methods. The result of lift and drainage velocity and temperature distributions are compared and presented graphically. The effects of various physical parameters are also discussed. Shah et al. [19] studied the solution of thin film flow of third grade fluid on moving inclined plane by using OHAM. Siddiqui et al. [20] investigated the thin film flow of a third grade fluid over an inclined plane. The non-linear equation of velocity field is solved by using OHAM and traditional perturbation method.

Based on the above motivation, the main goal of the present work is to venture further in the regime of Oldroyd-B fluid. More exactly, this article aims to study the unsteady MHD thin film flow of an Oldroyd-B fluid past an oscillating inclined belt using Optimal Homotopy Asymptotic Method (OHAM) and Homotopy Perturbation Method (HPM). These methods have been used successfully in the literature for the solutions of non-linear fluid problems. Marinca et al. [21–24] discussed the approximate solution of non-linear steady flow of fourth grade fluid by using OHAM. They noticed from the results that OHAM method is more effective and easy to use then other methods. Kashkari [25] studied the OHAM solution of nonlinear Kawahara equation. For comparison HPM, VHPM and VIM method is used but OHAM is more successful method. He [26–30] provided the fundamental introduction of HPM and solved the wave equation. Sanela et al. [31] solved nonlinear partial differential equations using HPM. Nofel [32] studied application of homotopy perturbation method for nonlinear differential equations. Ganji et al. [33] studied the solution of Blasius non differential equation using HPM. Anakira et al. [34] discussed the analytical solution of delay differential equation using OHAM. Mabood et al. [35,36] investigated the approximate solution of non-linear Riccati differential equation by using OHAM.

## Basic Equation

Let us consider the unsteady MHD incompressible flow over an inclined belt defined by the following equations

divV=0, (1)

$$\rho \frac{D\text{v}}{Dt}=div\text{T}+\rho g\text{sin}\theta +\text{J}\times \text{B},$$ (2)

Where **V** is the velocity vector of the fluid, *ρ* is the fluid density, $\frac{D}{Dt}$ is the material time derivative, and *g* is the external body force. Thus,the Lorentz force perunit volume is

$$\text{J}\times \text{B}=\left[0,\sigma B_{0}^{2}v,0\right],$$ (3)

where **B** = (0,*B*_0_,0) is the uniform magnatic filed, *B*_0_ is theapplied magnetic field and *σ* is the electrical conductivity.

The current density **J** is

$$\text{J}=\sigma \left(\text{E}+\text{V}\times \text{B}\right),\nabla \times \text{B}=\mu_{0}\text{J}.$$ (4)

Here, *μ*_0_ is the magnetic permeability, **E** is an electric field which is not considered in this study, and

$$\frac{D{\text{B}}_{1}}{Dt}=\frac{\partial {\text{B}}_{1}}{\partial t}+\left(\text{V}.\nabla \right){\text{B}}_{1}-\left(\nabla \text{V}\right){\text{B}}_{1}-{\text{B}}_{1}{\left(\nabla \text{V}\right)}^{T}.$$ (5)

The above model can be reduced to different types of fluid depend on *λ*_1_ (relaxation time) and *λ*_2_ (retardation time). In Eq (5), if *λ*_1_ = *λ*_2_ the fluid becomes viscous. When *λ*_2_ = 0, it becomes a Maxwell fluid and reduced to Oldroyd-B fluid when 0<*λ*_2_<*λ*_1_<1.

The cauchy stress tensor, **T** is

T=−pI+S, (6)

$$\text{S}+\lambda_{1}\frac{D\text{S}}{Dt}=\mu \left[1+\lambda_{2}\frac{D}{Dt}\right]{\text{A}}_{1},$$ (7)

$${\text{A}}_{0}=\text{I},{\text{A}}_{1}=\text{L}+{\text{L}}^{T},L=grad\text{V},$$ (8)

Where **S** is the extra tress tensor, *p* **I** is the isotropic stress, **A**_1_ is the Rivlin Ericksen stress tensor and *μ* is the viscosity cofficient.

## Formulation of the Problem

Let us consider a thin film flow of a non-Newtonian Oldroyd-B fluid on an oscillating inclined belt. The force of gravity will initiate the motion of a layer of liquid in the downward direction. The thickness, *δ*, of the of liquid layer is considered to be uniform. A uniform magnetic field is applied to the belt in the direction perpendicular to the fluid motion. The external electric field is not considered and the magnetic Reynolds number is negligible, which implies that the current is totally dependent on the induced electric field and the electric current flowing in the fluid does not affect the magnetic field. The induced magnetic field created by the fluid motion is very small compared to the applied magnetic field. Therefore, the Lorentz force term in Eq (2) is reduced to $-\sigma B_{0}{}^{2}\text{v},$ assuming that the flow is unsteady, laminar, incompressible, and pressure gradient is zero.

The velocity field is of the form

v=(v(y,t),0,0) and S=(y,t), (9)

subject to the boundary conditions

$$v\left(0,t\right)=V\text{cos}\omega t,\frac{\partial v\left(\delta ,t\right)}{\partial y}=0,$$ (10)

where *ω* is the frequency of the oscillating belt.

The momentum Eq (2) is reduced to

$$\rho \frac{\partial v}{\partial t}=-\frac{\partial p}{\partial x}+\frac{\partial S_{xy}}{\partial y}+\rho g\text{sin}\theta -\sigma B_{0}{}^{2}v,$$ (11)

$$\frac{\partial p}{\partial y}=\frac{\partial S_{yy}}{\partial y},$$ (12)

$$\frac{\partial p}{\partial z}=0.$$ (13)

It follows from (7) and (9) that

$$S_{xx}+\lambda_{1}\left[\frac{\partial S_{xx}}{\partial t}-2S_{xy}\frac{\partial v}{\partial y}\right]=-2\mu \lambda_{2}{\left[\frac{\partial v}{\partial y}\right]}^{2},$$ (14)

$$S_{xy}+\lambda_{1}\left[\frac{\partial S_{xy}}{\partial t}-S_{yy}\frac{\partial v}{\partial y}\right]=\mu \left(\frac{\partial v}{\partial y}\right)+\lambda_{2}\mu \left(\frac{\partial^{2}v}{\partial t\partial y}\right),$$ (15)

$$S_{yy}+\lambda_{1}\frac{\partial S_{yy}}{\partial t}=0,$$ (16)

then Eq (16) reduces to

$$S_{yy}=B\left(y\right)e^{-\frac{t}{\lambda_{1}}}.$$ (17)

Here *B*(*y*) is used as an arbitrary function. When *t*<0, then *S*_*yy*_ is reduced to zero, which demonstrates that *B*(*y*) must also be zero. Therefore, from the Eqs (11) and (15) and in the presence of zero pressure gradient, we obtain

$$\left(1+\lambda_{1}\frac{\partial }{\partial t}\right)\frac{\partial v}{\partial t}=v\left(1+\lambda_{2}\frac{\partial }{\partial t}\right)\frac{\partial^{2}v}{\partial y^{2}}-\frac{\sigma B_{0}{}^{2}}{\rho }\left(1+\lambda_{1}\frac{\partial }{\partial t}\right)v+\rho g\text{sin}\theta .$$ (18)

Introducing non-dimensional variables

$$v=\frac{\overset{⌣}{v}}{V},y=\frac{\overset{⌣}{y}}{\delta },t=\frac{\mu \overset{⌣}{t}}{\rho \delta^{2}},k_{1}=\frac{\lambda_{1}\mu }{\rho \delta^{2}},k_{2}=\frac{\lambda_{2}\mu }{\rho \delta^{2}},\omega =\frac{\overset{⌣}{\omega }\delta^{2}\rho }{\mu },m=\frac{\delta^{2}\rho g\text{sin}\theta }{\mu U},M=\frac{\sigma B_{0}^{2}\delta^{2}}{\mu },$$ (19)

where, *ω* is the oscillating parameter, *k*_1_ is the relaxation paramter, *k*_2_ is the retardation parameter, *m* is the gravitational parameter and *M* is the magnetic parameter.

By inserting the non-dimensional variables from Eq (19) into momentum Eq (18), boundary conditions Eq (10) and dropping bars we obtain:

$$\left(1+k_{1}\frac{\partial }{\partial t}\right)\frac{\partial v}{\partial t}=\left(1+k_{2}\frac{\partial }{\partial t}\right)\frac{\partial^{2}v}{\partial y^{2}}-M\left(1+k_{1}\frac{\partial }{\partial t}\right)v+m,$$ (20)

$$v\left(0,t\right)=\text{cos}\omega t,\frac{\partial v\left(1,t\right)}{\partial y}=0.$$ (21)

## Basic Idea of HPM

To illustrate the central concept of HPM to solve non-linear partial differential equation, we consider the following partial differential equation

D(v(y,t))−Q(y,t)=0,  B(v,t)=0, (22)

where *v*(*y*,*t*) is the unknown function, *Q*(*y*,*t*) is the known analytic function, *B* is the boundary operator, and *D* is the general differential operator which is expressed in linear part *L*(*v*(*y*,*t*)) and non-linear part *N*(*v*(*y*,*t*)) as

D(v(y,t))=L(v(y,t))+N(v(y,t)), (23)

Therefore Eq (22) can be written as

L(v(y,t))+N(v(y,t))−Q(y,t)=0, (24)

Using homotopic method, this can be defined as

$$H(v(y,t),q)=(1-q)\left[L(v(y,t))-L(v_{0}(y,t))\right]+q\left[Dv(y,t)-Q(y,t)\right].$$ (25)

We can also write Eq (25) as

$$H(v(y,t),q)=L(v(y,t))-L(v_{0}(y,t)+qL(v_{0}(y,t))+q\left[N(v(y,t))-Q(y,t)\right].$$ (26)

Here *q*ϵ[0,1] is the embedding parameter and *v*_*0*_(*y*,*t*) is the initial approximation of Eq (22) satisfying the boundary condition.

From Eq (26)

$$q=0,\text{ H(v(y,t,),0)}=L(v(y,t))-L(v_{0}(y,t))=0,$$ (27)

q=1,   H(v(y,t,),1)=D(v(y,t))−Q(y,t)=0. (28)

By the variation of *q* from 0 to 1, *v*(*y*,*t*,*q*) changes from *v*_0_(*y*,*t*) to *v*(*y*,*t*) which is called deformation, *L*(*v*(*y*,*t*))-*L*(*v*_0_(*y*,*t*)) and *D*(*v*(*y*,*t*))-*Q*(*y*,*t*) are called homotropic.

Approximation solution of Eq (22) can be expressed as a series of the power of *q* as

$$v(y,t)=v_{0}(y,t)+qv_{1}(y,t)+q^{2}v_{2}(y,t)+..$$ (29)

Introducing *q* = 1 in Eq (29), the approximate solution of Eq (22) becomes

$$v(y,t)={\text{lim}}_{q\to 0}=v_{0}(y,t)+v_{1}(y,t)+v_{2}(y,t)+..$$ (30)

## Basic Theory of OHAM

Here we will discuss the primary concept of OHAM, considering a general partial differential equation of the form

$$L(v(y,t))+N(v(y,t))+G(y,t)=0,\text{ B }\left(v(y,t),\frac{\partial v(y,t)}{\partial y}\right)=0,\text{ y}\in \Omega \text{,}$$ (31)

where *L* is the linear operator, *N* is the non-linear operator, *G* is the known function, *v*(*y*,*t*) is the unknown function, *y* is the spatial independent variable, *t* is time independent variable and *B* is the boundary operator, and Ω is the domain of the problem.

According to the basic theory of OHAM, the optimal homotopy *ψ*(*y*,*t*,*p*):*ψ*×[0,1]→*R*, needs to satisfy the following equation

$$\left[1-p\right]\left[L\psi \left(y,t,p\right)+G\left(y,t\right)\right]=\left[\begin{array}{l} H\left(p,c_{i}\right)\left[L\psi \left(y,t,p\right)+G\left(y,t\right)+N\psi \left(y,t,p\right)\right],\\ B\left(\psi \left(y,t,p\right),\frac{\partial \psi \left(y,t,p\right)}{\partial y}\right)=0 \end{array}\right].$$ (32)

Here, *p* is the embedding parameter and *p*ϵ[0,1], *H*(*p*) is the non-zero auxiliary function for *p*≠0, *H*(0) = 0. From Eq (31) we can clearly write

p=0⇒H(ψ(y,t,0),0)=H(0)[Lψ(y,t)+Gψ(y,t,p)]=0, (33)

$$p=1\Rightarrow H(\psi \left(y,t,1\right),1)=H(1,c_{i})\left[L\psi \left(y,t\right)+G\psi \left(y,t,p\right)+N\psi \left(y,t,p\right)\right]=0,$$ (34)

Clearly it holds that when *p* = 0,*ψ*(*y*,*t*,*0*) = *v*_0_(*y*,*t*) and when *p* = 1 then *ψ*(*y*,*t*,*1*) = *v*(*y*,*t*), We obtain *v*_0_(*y*,*t*) by inserting *p* = *0* in Eq (31)

Lψ(y,t)+Gψ(y,t,p)=0. (35)

Here we select the auxiliary function as:

$$H(p,c_{i})=pc_{1}+p^{2}c_{2}+p^{3}c_{3}\ldots \ldots \ldots ,$$ (36)

where *c*_1_,*c*_*2*_,*c*_3_ are called convergence controle parameters and will be determined accordingly.

To find the approximate solution, we expand the unknown function *ψ*(*y*,*t*,*p*)as

$$\psi \left(y,t,p,c_{i}\right)=v_{0}(y,t)+\sum_{k\geq 1}v_{k}\left(y,t,p,c_{i}\right)p^{k}.$$ (37)

By inserting Eq (37) into Eq (31) and equating the identical power of *p*, we obtain the zero, first and second order problem, *v*_0_(*y*,*t*), *v*_1_(*y*,*t*) and *v*_2_(*y*,*t*), so the governing equation is:

$$L\left(v_{1}\left(y,t\right)\right)+G\left(y,t\right)=c_{1}N_{0}\left(v_{0}\left(y,t\right)\right),\;B\left(\frac{\partial v_{1}\left(y,t\right)}{\partial y}\right)=0,$$ (38)

$$L\left(v_{2}\left(y,t\right)\right)-L\left(v_{1}\left(y,t\right)\right)=\left[\begin{array}{l} c_{2}N_{0}\left(v_{0}\left(y,t\right)\right)+c_{1}\left[L\left(v_{1}\left(y,t\right)\right)+N_{1}\left(v_{0}\left(y,t\right),v_{1}\left(y,t\right)\right)\right],\\ B\left(v_{2}\left(x\right),\frac{\partial v_{2}\left(y,t\right)}{\partial y}\right) \end{array}\right]=0.$$ (39)

The general governing equations for *u*_k_(*y*,*t*) are given by

$$L\left(v_{k}\left(y,t\right)\right)-L\left(v_{k-1}\left(y,t\right)\right)=\left[\begin{array}{l} c_{k}N_{0}\left(v_{0}\left(y,t\right)\right)+\sum_{i=1}^{k-1}c_{i}\left[\begin{array}{l} L\left(v_{k-i}\left(y,t\right)\right)+\\ N_{k-1}\left(v_{0}\left(y,t\right),v_{1}\left(y,t\right)\ldots \ldots v_{k-i}\left(y,t\right)\right) \end{array}\right],\\ k=2,3,\ldots ,B\left(v_{k}\left(y,t\right),\frac{\partial v_{k}\left(y,t\right)}{\partial y}\right)=0, \end{array}\right]$$ (40)

Here *N*_*m*_(*v*_0_(*y*,*t*),*v*_1_(*y*,*t*)….*v*_m-1_(*y*,*t*)) is the coefficient of *p* ^*m*^, in the expansion of *Nψ*(*y*,*t*,*p*).

$$N\left(\psi \left(y,t,p,c_{i}\right)\right)=N_{0}\left(v_{0}\left(y,t\right)\right)+\sum_{m=1}^{\infty }N_{m}\left(v_{0}\left(y,t\right),v_{1}\left(y,t\right)\ldots .v_{m}\left(y,t\right)\right)p^{m}.$$ (41)

The convergence of the series in Eq (36) depend upon the convergence controle parameters *c*_1_,*c*_2_,… If it converges at *p* = 1, then the *mth* order approximation *v* is

$$v\left(y,c_{1},c_{2}\ldots .c_{m}\right)=v_{0}\left(y,t\right)+\sum_{i=1}^{m}v_{i}\left(y,c_{1},c_{2}\ldots .c_{i}\right).$$ (42)

Inserting Eq (42) into Eq (32), the residual is obtained as:

$$R\left(y,t,c_{i}\right)=L\left(v\left(y,t,c_{i}\right)\right)+G\left(y,t\right)+N\left(v\left(y,t,c_{i}\right)\right),i=1,2..m$$ (43)

Numerous methods like Ritz Method, Method of Least Squares, Galerkin’s Method and Collocation Method are used to find the optimal values of *c*_*i*_,*i* = *1*,*2*,*3*,*4*… We apply the Method of Least Squares in our problem as given below:

$$j\left(c_{1},c_{2},\ldots c_{n}\right)=\int_{a}^{b}R^{2}\left(y,t,c_{1},c_{2},\ldots c_{m}\right)dy,$$ (44)

where *a* and *b* are the constant values taking from domain of the problem.

Auxiliary constants *c*_1_,*c*_2_,…*c*_*n*_ can be identified from:

$$\frac{\partial j}{\partial c_{1}}=\frac{\partial j}{\partial c_{1}}=\ldots =0.$$ (45)

Finally, from these convergence controle parameters, the approximate solution is well-determined.

## HPM Solution

By applying HPM method to Eq (20) with boundary condition (21), we obtain zero, first and second component problems of the velocity profile.

The component problems of velocity profile are

$$p^{0}:\frac{\partial^{2}v_{0}\left(y,t\right)}{\partial y^{2}}=-m,$$ (46)

$$p^{1}:\frac{\partial^{2}v_{1}\left(y,t\right)}{\partial y^{2}}=2m-Mv_{0}-Mk_{1}\frac{\partial v_{0}}{\partial t}-k_{1}\frac{\partial^{2}v_{0}}{\partial t^{2}}+2\frac{\partial^{2}v_{0}}{\partial y^{2}}+k_{2}\frac{\partial }{\partial t}\left(\frac{\partial^{2}v_{0}}{\partial y^{2}}\right),$$ (47)

$$p^{2}:\frac{\partial^{2}v_{2}\left(y,t\right)}{\partial y^{2}}=-Mv_{1}-\left(1+Mk_{1}\right)\frac{\partial v_{1}}{\partial t}-k_{1}\frac{\partial^{2}v_{1}}{\partial t^{2}}+2\frac{\partial^{2}v_{1}}{\partial y^{2}}+k_{2}\frac{\partial }{\partial t}\left(\frac{\partial^{2}v_{1}}{\partial y^{2}}\right).$$ (48)

Solutions of Eqs (22–24) using boundary condition in Eq (20) is

$$v_{0}\left(y,t\right)=\text{cos}\left[t\omega \right]+\left[\frac{m}{2}-\text{cos}\left[t\omega \right]\right]y-\left[\frac{m}{2}\right]y^{2},$$ (49)

$$\left(y,t\right)=\left[\begin{array}{l} \left[\frac{1}{3}\left(M-\omega^{2}k_{1}\right)\text{cos}\left[t\omega \right]-\frac{\omega }{3}\left(1+Mk_{1}\right)\text{sin}\left[t\omega \right]\right]y-\\ {}[\frac{1}{2}\left(M-k_{1}\omega^{2}\right)\text{cos}\left[t\omega \right]-\frac{\omega }{2}\left(1+Mk_{1}\right)\text{sin}\left[t\omega \right]]y^{2}-\\ \frac{1}{12}\left[mM-2\left(M-\omega^{2}k_{1}\right)\text{cos}\left[t\omega \right]+2\omega \left(1+Mk_{1}\right)\text{sin}\left[t\omega \right]\right]y^{3}\\ +\frac{mM}{24}\left(y+y^{4}\right) \end{array}\right]\;,$$ (50)

$$v_{2}\left(y,t\right)=\left[\begin{array}{l} \left[\begin{array}{l} \frac{1}{45}(2\omega^{3}k_{1}+2M\omega^{3}k_{1}{}^{2}-15k_{2}M\omega +15k_{1}k_{2}\omega^{3}-30\omega -\\ 2M\omega -30M\omega k_{1}-2M^{2}\omega k_{1})sin\left[t\omega \right]+\left[\frac{mM}{12}+\frac{mM^{2}}{240}\right] \end{array}\right]y+\\ \left[\begin{array}{l} \frac{1}{45}(30M+M^{2}-\omega^{2}-30\omega^{2}k_{1}-4M\omega^{2}k_{1}-M^{2}\omega^{2}k_{1}{}^{2}+\\ \omega^{4}k_{1}{}^{2}-15\omega^{2}k_{2}-15M\omega^{2}k_{1}k_{2})\text{cos}\left[t\omega \right] \end{array}\right]y+\\ \frac{1}{2}[(2k_{1}\omega^{2}+\omega^{2}k_{2}+M\omega^{2}k_{1}k_{2}-2M)\text{cos}\left[t\omega \right]+\\ \left(2\omega +2M\omega k_{1}+M\omega k_{2}-\omega^{3}k_{1}k_{2}\right)\text{sin}\left[t\omega \right]-m]y^{2}+\frac{1}{18}[(M-M^{2}+\omega^{2}\\ -6\omega^{2}k_{1}+4M\omega^{2}k_{1}+M^{2}\omega^{2}k_{1}{}^{2}-\omega^{4}k_{1}{}^{2}-3\omega^{2}k_{2}-3M\omega^{2}k_{1}k_{2})\text{cos}\left[t\omega \right]+\\ (2M^{2}\omega k_{1}-2\omega^{3}k_{1}-2M\omega^{3}k_{1}{}^{2}-3M\omega k_{2}+3\omega^{3}k_{1}k_{2}-6\omega +2M\omega -6M\omega k_{1})\text{sin}\left[t\omega \right]\\ -\frac{mM}{6}-\frac{mM^{2}}{144}]y^{3}+[\frac{mM}{12}+\frac{1}{24}(M^{2}-\omega^{2}-M^{2}\omega^{2}k_{1}{}^{2}+\omega^{4}k_{1}{}^{2}-4M\omega^{2}k_{1})\text{cos}\left[t\omega \right]\\ +\frac{1}{12}\left(\omega^{3}k_{1}+k_{1}{}^{2}M\omega^{3}-M\omega -M^{2}\omega k_{1}\right)\text{sin}\left[t\omega \right]]y^{4}+\\ {}[\frac{mM^{2}}{240}+\frac{1}{120}\left(\omega^{2}-M^{2}+4M\omega^{2}k_{1}+M^{2}\omega^{2}k_{1}{}^{2}-\omega^{4}k_{1}{}^{2}\right)\text{cos}\left[t\omega \right]+\\ \frac{1}{60}(M\omega +M^{2}\omega k_{1}-\omega^{3}k_{1}-M\omega^{3}k_{1}{}^{2})\text{sin}\left[t\omega \right]]y^{5}-\left(\frac{mM^{2}}{720}\right)y^{6} \end{array}\right],$$ (51)

The series solutions of velocity profile up to second component is

$$v\left(y,t\right)=v_{0}\left(y,t\right)+v_{1}\left(y,t\right)+v_{2}\left(y,t\right),$$ (52)

$$v\left(y,t\right)=\left[\begin{array}{l} \text{cos}\left[t\omega \right]+[\frac{mM}{8}+\frac{m}{2}+\frac{mM^{2}}{240}+\frac{1}{45}(45M+M^{2}-\omega^{2}-45\omega^{2}k_{1}-4M\omega^{2}k_{1}-\\ M^{2}\omega^{2}k_{1}{}^{2}+\omega^{4}k_{1}{}^{2}-15\omega^{2}k_{2}-15M\omega^{2}k_{1}k_{2}-45)\text{cos}\left[t\omega \right]+\frac{1}{45}(2\omega^{3}k_{1}+\\ 2M\omega^{3}k_{1}{}^{2}-15k_{2}M\omega +15k_{1}k_{2}\omega^{3}-45\omega -2M\omega -45M\omega k_{1}-2M^{2}\omega k_{1})\text{sin}\left[t\omega \right]]y+\\ \frac{1}{2}[(3k_{1}\omega^{2}+\omega^{2}k_{2}+M\omega^{2}k_{1}k_{2}-3M)\text{cos}\left[t\omega \right]+\left(3\omega +3M\omega k_{1}+M\omega k_{2}-\omega^{3}k_{1}k_{2}\right)\\ \text{sin}\left[t\omega \right]-m]y^{2}+\frac{1}{18}[(4M-M^{2}+\omega^{2}-9\omega^{2}k_{1}+4M\omega^{2}k_{1}+M^{2}\omega^{2}k_{1}{}^{2}-\omega^{4}k_{1}{}^{2}-3\omega^{2}k_{2}-\\ 3M\omega^{2}k_{1}k_{2})\text{cos}\left[t\omega \right]+(2M^{2}\omega k_{1}-2\omega^{3}k_{1}-2M\omega^{3}k_{1}{}^{2}-3M\omega k_{2}+3\omega^{3}k_{1}k_{2}-9\omega +\\ 2M\omega -9M\omega k_{1})\text{sin}\left[t\omega \right]-\frac{mM}{4}-\frac{mM^{2}}{144}]y^{3}+[\frac{mM}{8}+\frac{1}{24}(M^{2}-\omega^{2}-M^{2}\omega^{2}k_{1}{}^{2}+\\ \omega^{4}k_{1}{}^{2}-4M\omega^{2}k_{1})\text{cos}\left[t\omega \right]+\frac{1}{12}\left(\omega^{3}k_{1}+k_{1}{}^{2}M\omega^{3}-M\omega -M^{2}\omega k_{1}\right)\text{sin}\left[t\omega \right]]y^{4}+\\ {}[\frac{mM^{2}}{240}+\frac{1}{120}\left(\omega^{2}-M^{2}+4M\omega^{2}k_{1}+M^{2}\omega^{2}k_{1}{}^{2}-\omega^{4}k_{1}{}^{2}\right)\text{cos}\left[t\omega \right]+\frac{1}{60}(M\omega +\\ M^{2}\omega k_{1}-\omega^{3}k_{1}-M\omega^{3}k_{1}{}^{2})\text{sin}\left[t\omega \right]]y^{5}-\left(\frac{mM^{2}}{720}\right)y^{6} \end{array}\right]$$ (53)

## OHAM Solution

In this section, we applied OHAM method to Eq (20) with boundary condition in Eq (21) and study component problems of zero, first and second.

The component problems of velocity profile are

$$p^{0}:\frac{\partial^{2}v_{0}\left(y,t\right)}{\partial y^{2}}+m=0,$$ (54)

$$p^{1}:\;\frac{\partial^{2}v_{1}\left(y,t\right)}{\partial y^{2}}=\left[\begin{array}{l} m\left(1+c_{1}\right)-Mc_{1}v_{0}-c_{1}\left(1+Mk_{1}\right)\frac{\partial v_{0}}{\partial t}-k_{1}c_{1}\frac{\partial^{2}v_{0}}{\partial t^{2}}+\\ \left(1+c_{1}\right)\frac{\partial^{2}v_{0}}{\partial y^{2}}+k_{2}c_{1}\frac{\partial }{\partial t}\left(\frac{\partial^{2}v_{0}}{\partial y^{2}}\right) \end{array}\right]$$ (55)

Solutions to Eqs (54–55) using boundary condition in Eq (21) are

$$v_{0}\left(y,t\right)=\text{cos}\left[t\omega \right]+\left[\frac{m}{2}-\text{cos}\left[t\omega \right]\right]y-\left[\frac{m}{2}\text{cos}\left[t\omega \right]\right]y^{2},$$ (56)

$$v_{1}\left(y,t\right)=\left[\begin{array}{l} {}[\frac{1}{24}\left(mM+8\left(M-\omega^{2}k_{1}\right)\text{cos}\left[t\omega \right]-8\omega \left(1+Mk_{1}\right)\text{sin}\left[t\omega \right]\right)y-\\ \frac{1}{2}(\left(M-\omega^{2}k_{1}\right)\text{cos}\left[t\omega \right]-\omega \left(1+k_{1}M\right)\text{sin}\left[t\omega \right])y^{2}-\\ \frac{1}{12}\left(mM-\left(M-k_{1}\omega^{2}\right)\text{cos}\left[t\omega \right]+2\omega \left(1+Mk_{1}\right)\text{sin}\left[t\omega \right]\right)y^{3}+\left(\frac{mM}{24}\right)y^{4}] \end{array}\right]$$ (57)

The second component solution for velocity is too bulky, therefore, only graphical representations up to second order are given.

The series solutions of velocity profile is obtained as

$$v\left(y,t\right)=v_{0}\left(y,t\right)+v_{1}\left(y,t\right)+v_{2}\left(y,t\right)$$ (58)

The values of *c*_*i*_ for the velocity components are *c*_1_ = -1.093756464,*c*_2_ = 03004259427.

## Results and Discussion

**Fig 1:**
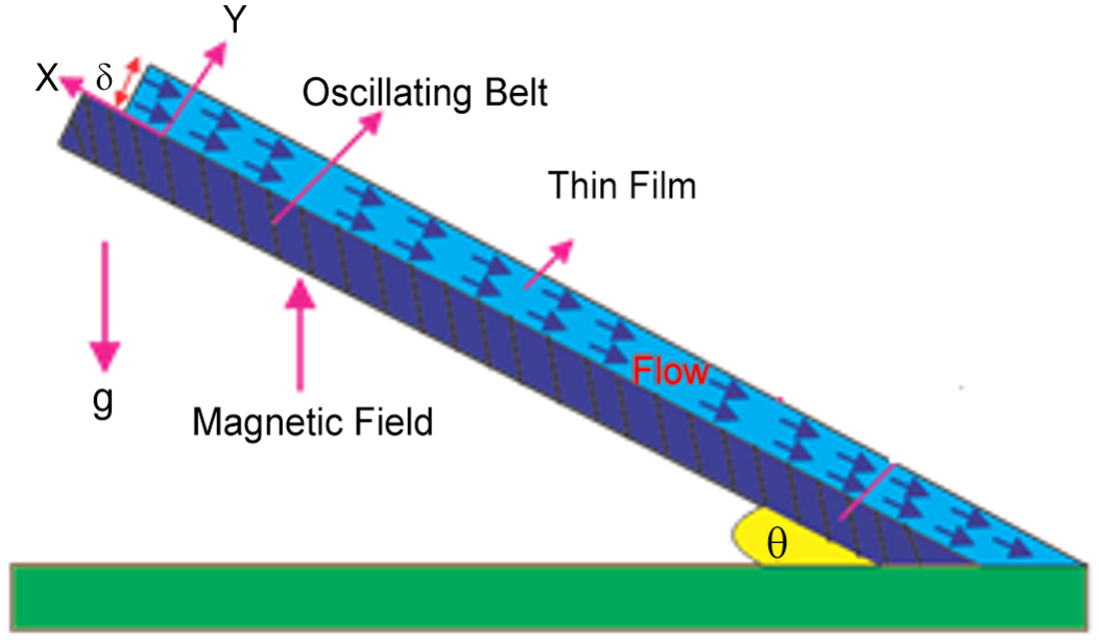
Geometry of the problem.

**Fig 2:**
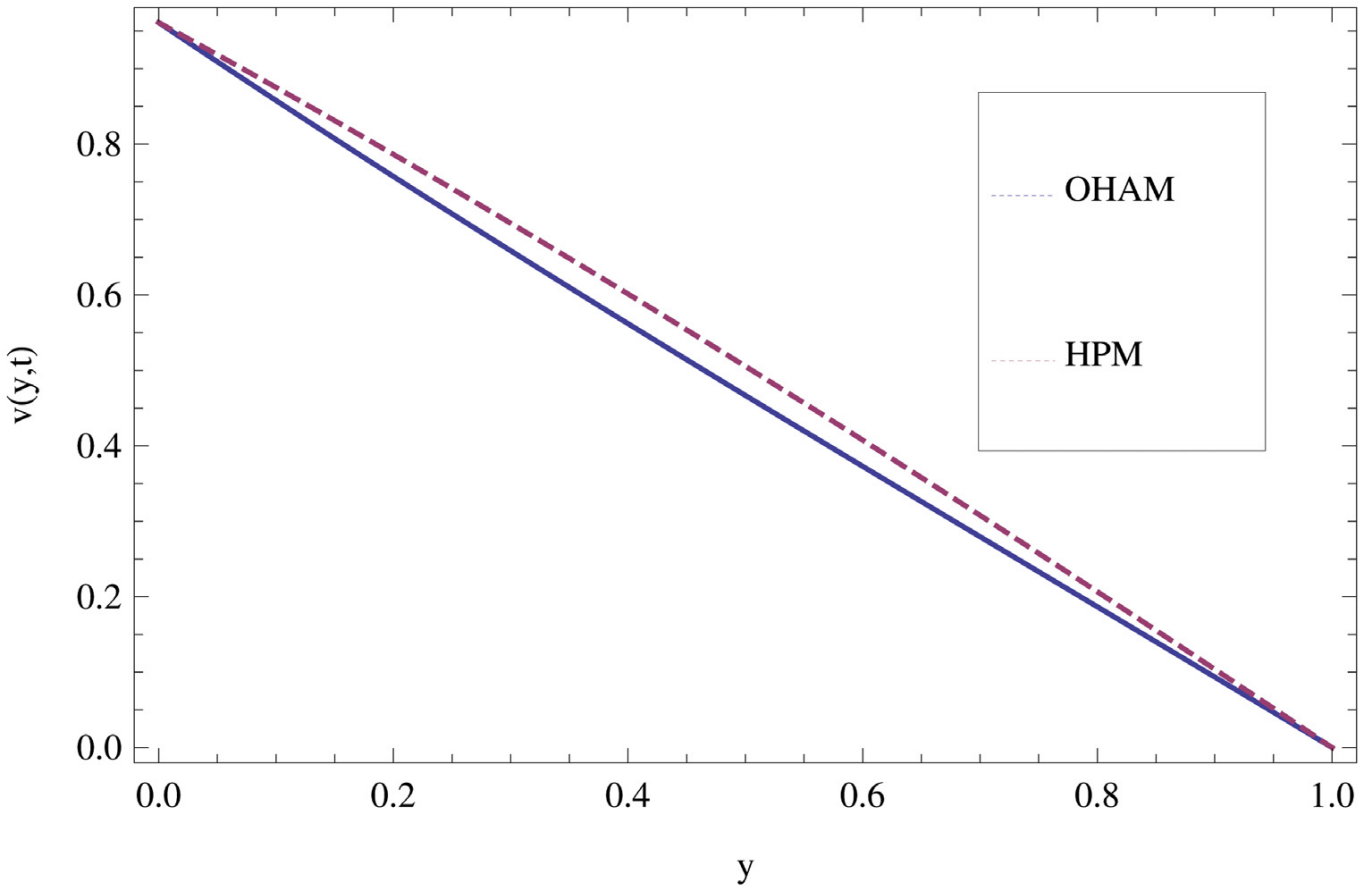
Comparison of OHAM and HPM solutions for velocity profile by when *ω* = 0.2,*m* = 0.4,*M* = 0.5,*t* = 5,*k*_1_ = 0.6,*k*_2_ = 0.3.

**Fig 3:**
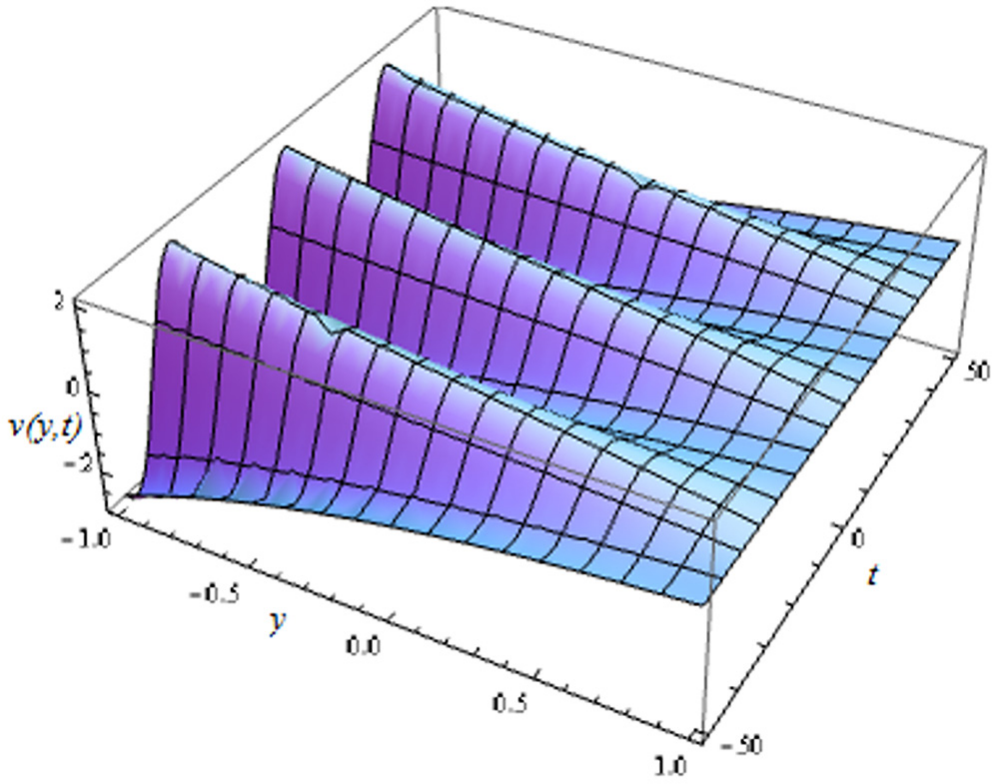
Influence of different time level, *ωt*ϵ[0,6*π*] on velocity profile.

**Fig 4:**
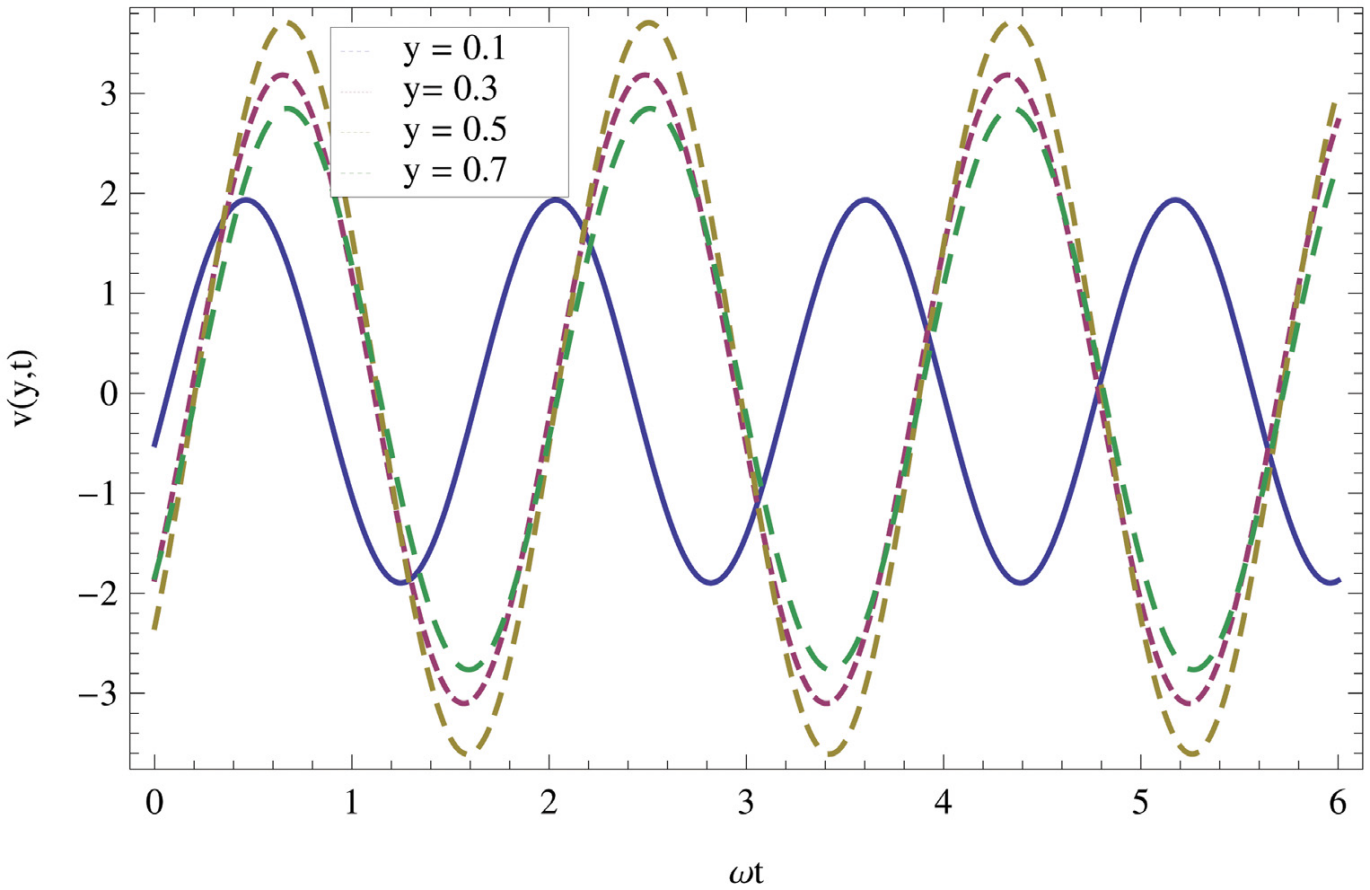
Velocity distribution of fluid for different time level when *ω* = 0.2,*m* = 0.1,*M* = 0.2,*t* = 5,*k*_1_ = 0.5,*k*_2_ = 0.3.

**Fig 5:**
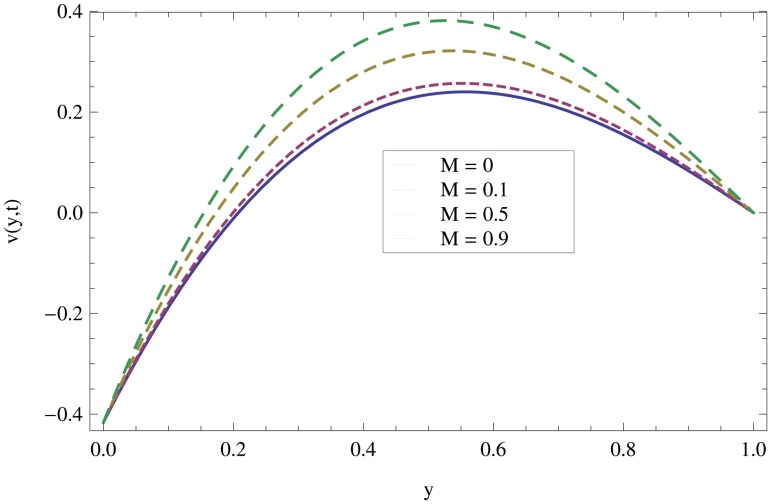
Effect of magnetic parameter *M* on the velocity profile when *ω* = 0.2,*y* = 0.5,*m* = 0.2,*t* = 7,*k*_1_ = 0.5,*k*_2_ = 0.3.

**Fig 6:**
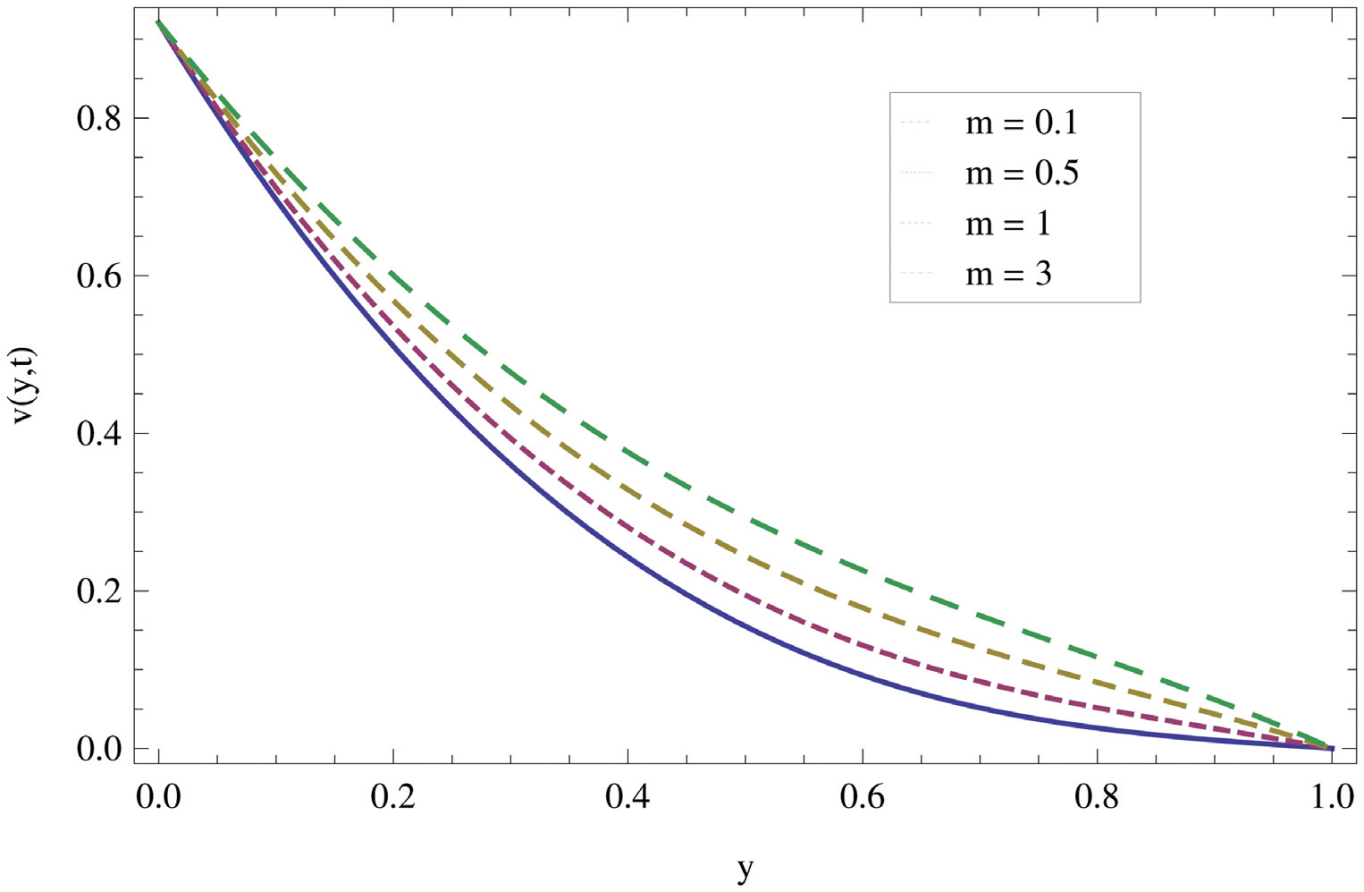
Effect of gravitational parameter when *y* = 0.4,*M* = 0.3,*t* = 10,*k*_1_ = 0.5,*k*_2_ = 0.3.

**Fig 7:**
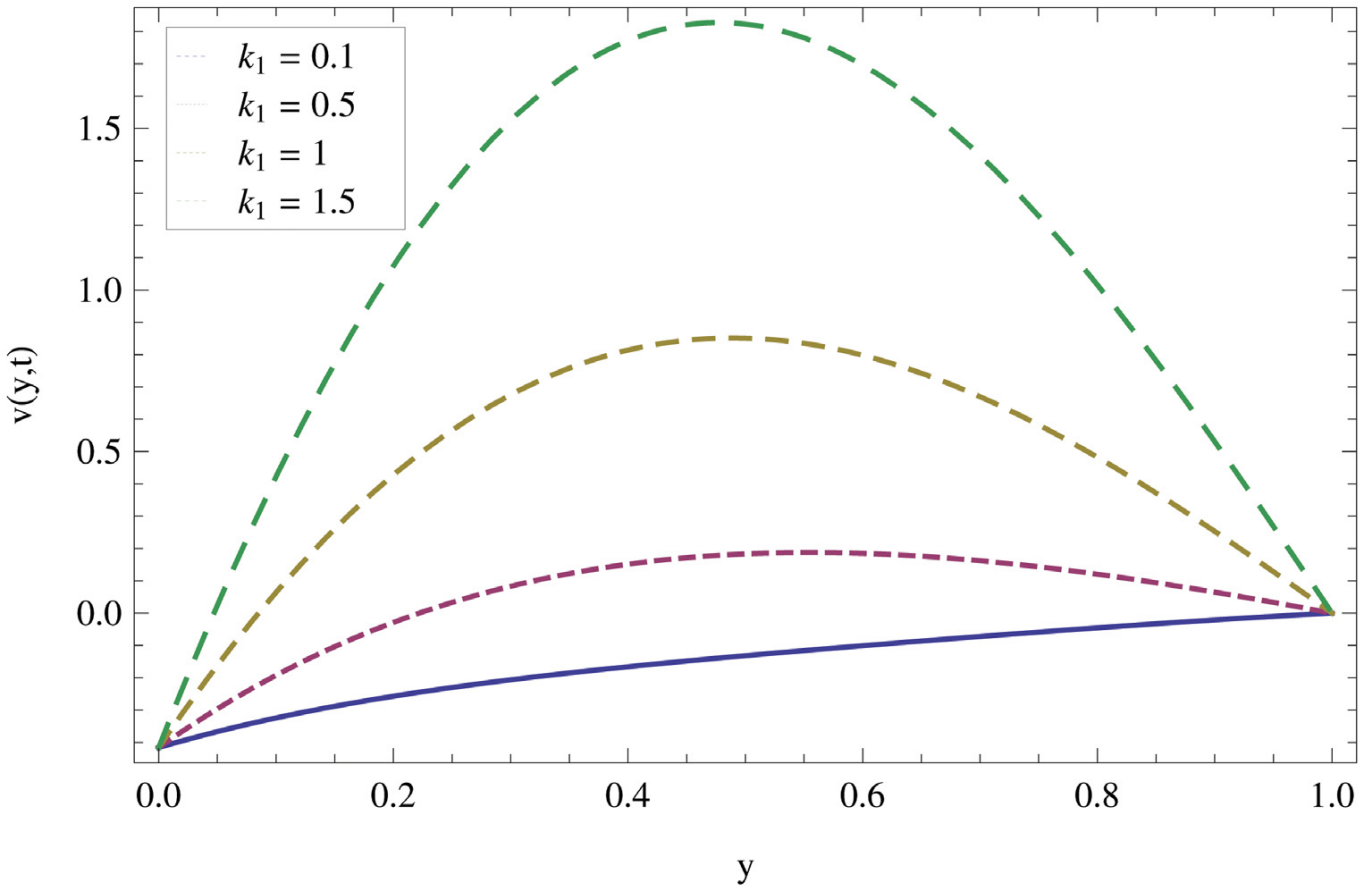
Effect of non-Newtonian parameter *k*_*2*_ on velocity profiles when *y* = 0.4,*M* = 0.3,*t* = 1,*k*_2_ = 0.3.

**Fig 8:**
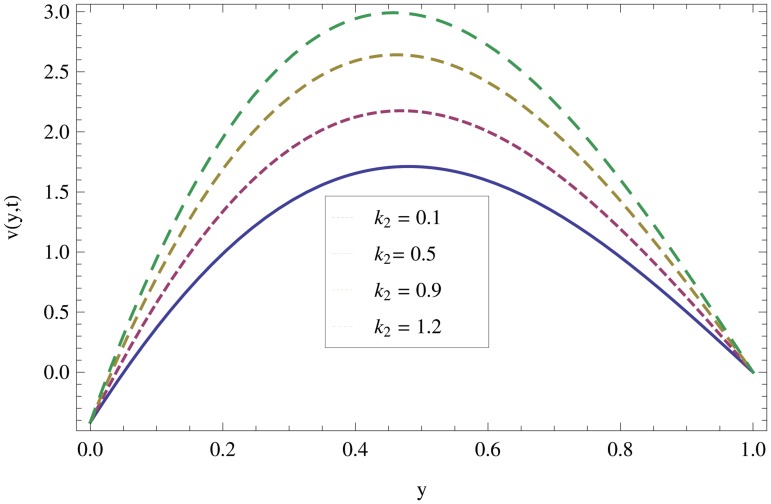
Effect of non-Newtonian parameter *k*_*2*_ on velocity profiles when *ω* = 0.2,*y* = 0.4,*M* = 0.3,*t* = 5,*k*_1_ = 0.5.

**Fig 9:**
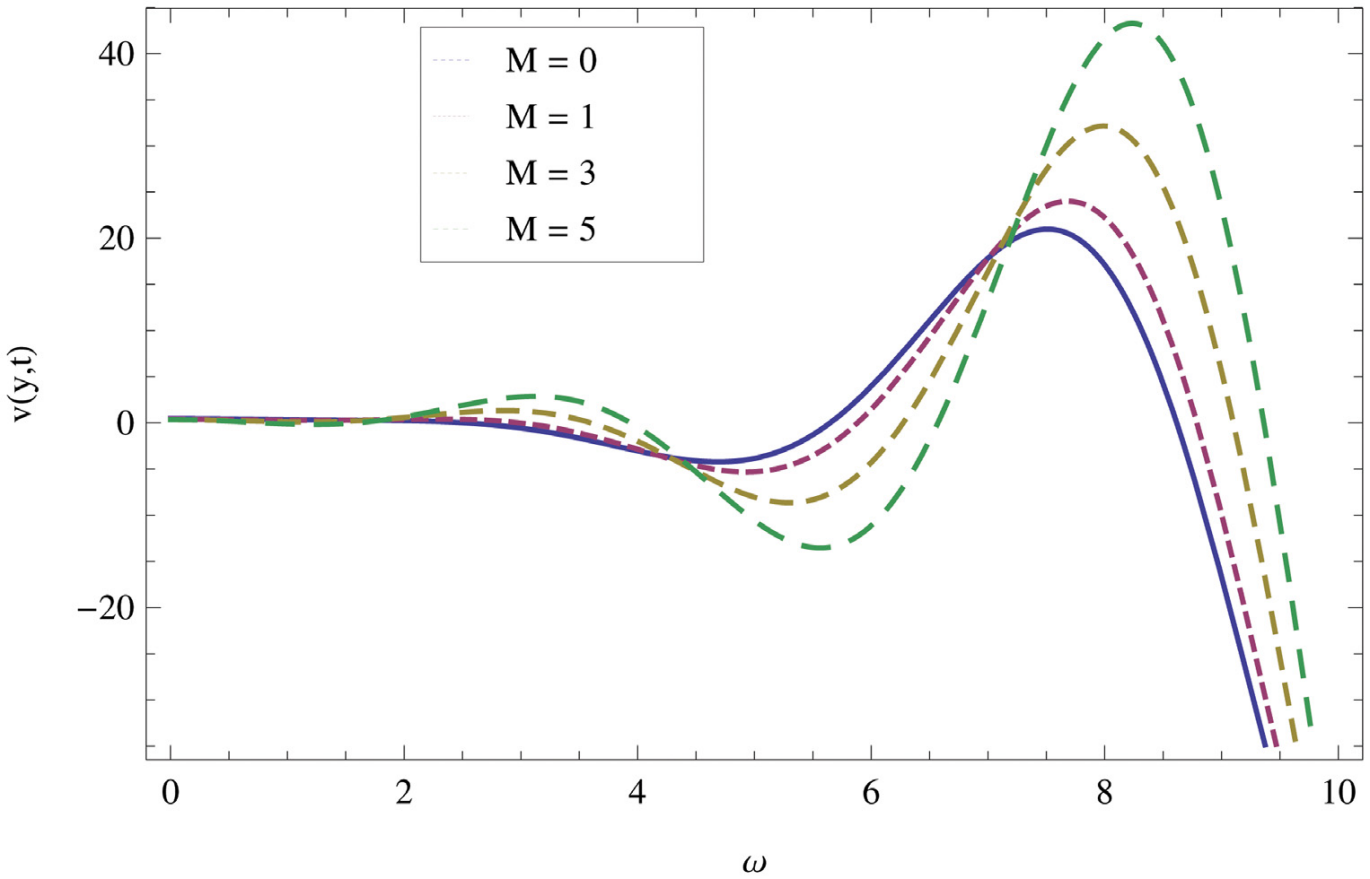
Effect of magnetic parameter *M* on the velocity profile when *ω* = 0.2,*y* = 0.5,*m* = 0.2,*t* = 5,*k*_2_ = 0.3.

**Fig 10:**
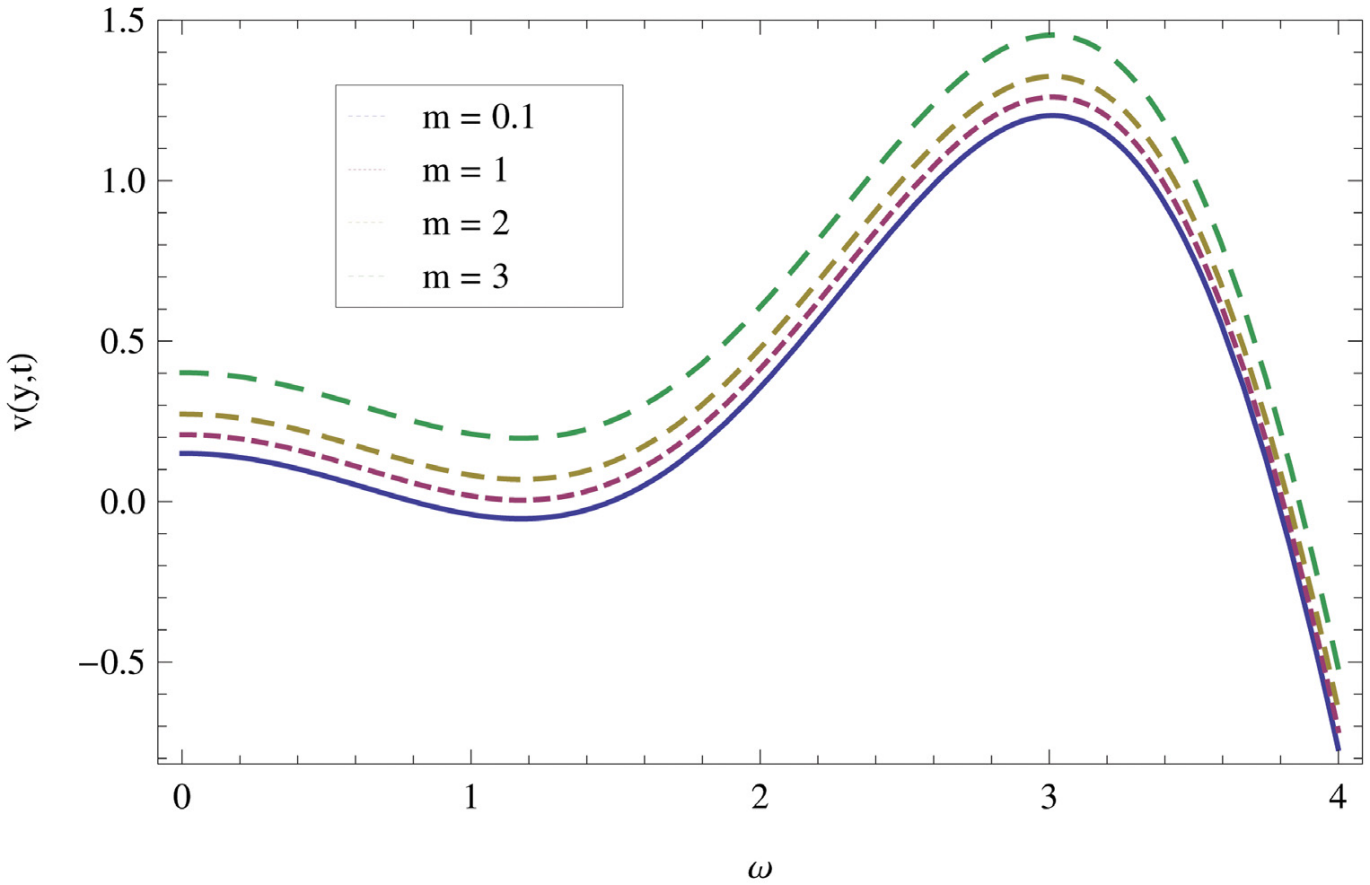
Effect of gravitational parameter on the velocity when *ω* = 0.2,*y* = 0.5,*m* = 0.2,*t* = 5,*k*_2_ = 0.3.

**Fig 11:**
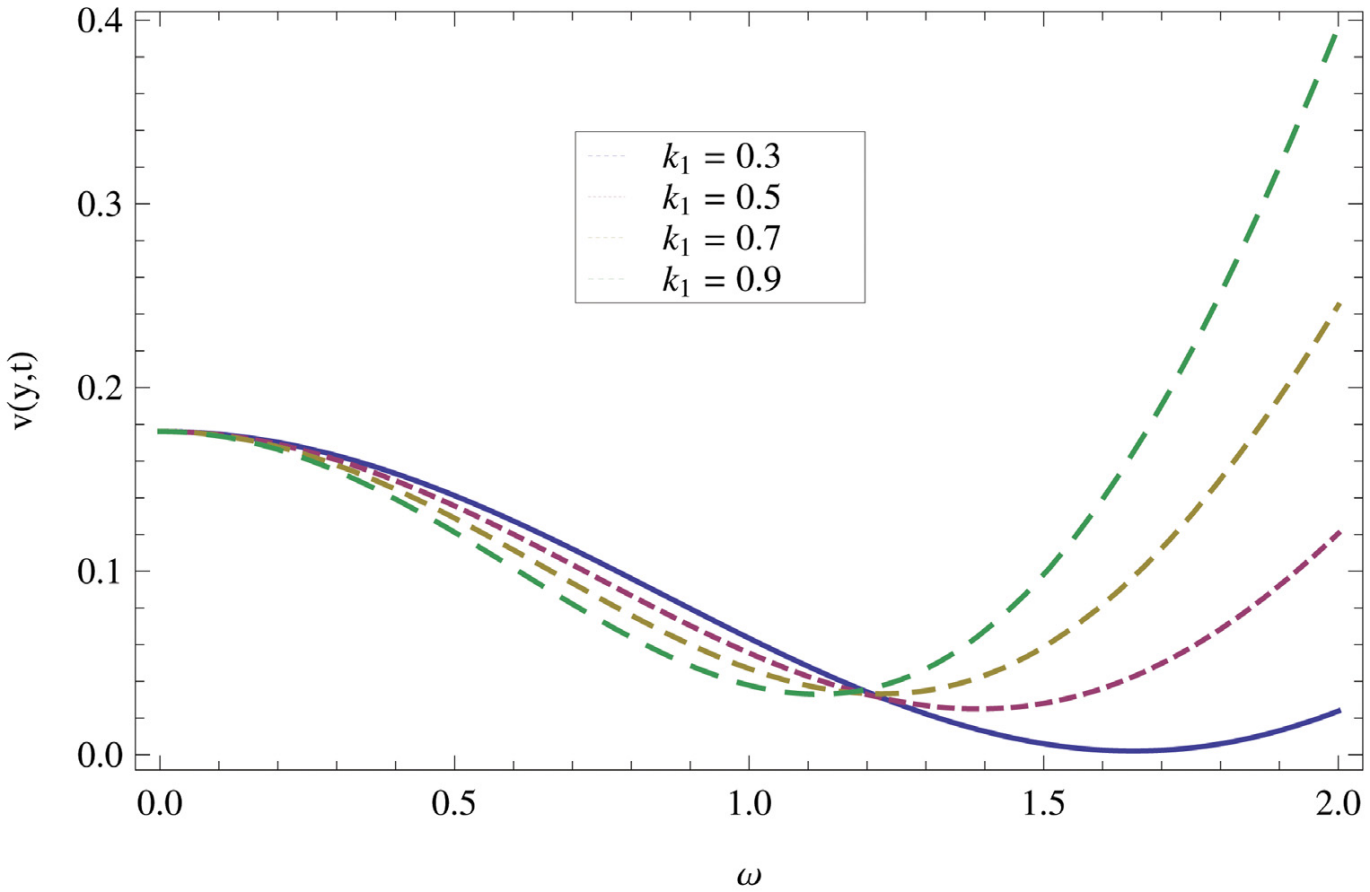
Effect of non-Newtonian parameter *k*_*1*_ on velocity profiles when *ω* = 0.2,*y* = 0.4,*M* = 0.3,*t* = 1,*k*_2_ = 0.1.

**Fig 12:**
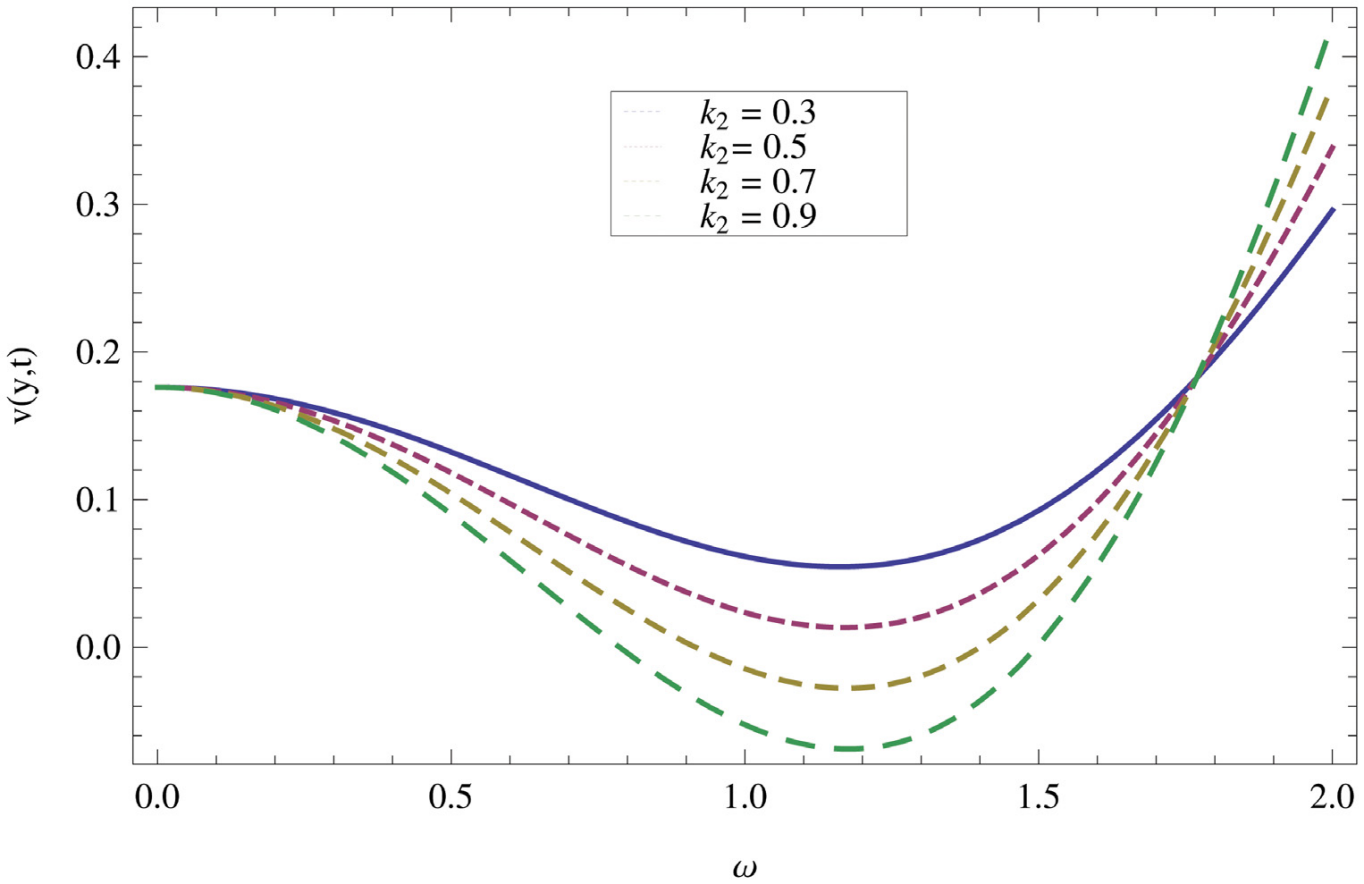
Effect of non-Newtonian parameter *k*_*2*_ on velocity profiles when *ω* = 0.2,*y* = 0.4,*M* = 0.3,*t* = 1,*k*_1_ = 0.5.

Unsteady MHD thin film flow of an Oldroyd-B fluid over an oscillating inclined belt has been examined. The governing partial differential equations for velocity are analytically solved by using OHAM and HPM methods. Both of these results are compared. It is found that these results are in excellent agreement. In tables 1 and 2, we calculated the numerical comparisons of OHAM and HPM. Absolute errors of both methods are also calculated. Fig 1 shows the physical configuration of the problem. The graphical comparison of OHAM and HPM solutions is shown in Fig 2 by taking different values of physical parameters. Figs (3–12) are plotted in order to observe the influence of different parameters on the velocity profiles. All results for the Oldroyd-B fluid near the belt are illustrated in the *y*-coordinate only for a selected domain *y*ϵ[0,1]. The effect of first three periods, *ω* = 0.2,*y* = 0.4,*M* = 0.3,*t* = 1,*k*_2_ = 0.1. are used to study the thin layer near the belt as shown in Fig (3). Clearly, due to the no-slip condition, the fluid near the belt oscillates jointly with the belt in the same period. The velocity amplitude raises gradually towards the surface of the fluid layer. The effect of transverse magnetic field on velocity is studied in Fig 4. Transverse magnetic field restricts the shearing and forming a thinner boundary layer near the belt. Due to this reason, the speed of flow increases towards the free surface of the belt. Fig 5 shows an increase in the fluid velocity when gravitational parameter *m* increases. Actually, it is due to friction which appears greater near the belt and smaller at the surface of the fluid. The effects of *k*_1_ (relaxation time parameter) and *k*_2_ (retardation time parameter) are shown Figs (7 and 8). Increase in these parameters increases the velocity profile.

**Table 1:** Comparison of OHAM and HPM for the velocity profile, when *ω* = 0.2,*m* = 0.1,*M* = 0.2,*t* = 5,*k*_1_ = 0.5,*k*_2_ = 0.3.

*x*	OHAM	ADM	Absolute Error
0.0	0.540302305	0.540302305	0
0.1	0.488777043	0.4908529494	2.07×10^−3^
0.2	0.436764196	0.440218095	3.65×10^−3^
0.3	0.384264222	0.3889869477	4.72×10^−3^
0.4	0.331261762	0.3365309755	5.26×10^−3^
0.5	0.277726280	0.2830412583	5.31×10^−3^
0.6	0.223612690	0.2285100876	4.89×10^−3^
0.7	0.168861963	0.1729348866	4.07×10^−3^
0.8	0.113401727	0.1163183844	2.91×10^−3^
0.9	0.057146847	0.0586687961	1.52×10^−3^
1.0	-4.16×10^−17^	-2.23×10^−17^	1.93×10^−17^

**Table 2:** Comparison of OHAM and HPM for the velocity profile, when *ω* = 0.2,*m* = 0.1,*M* = 0.2,*t* = 1,*k*_1_ = 0.5,*k*_2_ = 0.3

*x*	OHAM	ADM	Absolute Error
0.0	0.921061	0921061	0
0.1	0.823557	0.832988	9.43×10^−3^
0.2	0.728009	0.744052	1.60×10^−3^
0.3	0.634182	0.654252	2.01×10^−2^
0.4	0.541806	0.563578	2.17×10^−2^
0.5	0.4505886	0.72011	2.14×10^−2^
0.6	0.360202	0.37953	1.93×10^−2^
0.7	0.270315	0.286109	1.57×10^−2^
0.8	0.180568	0.191726	1.11×10^−2^
0.9	0.0905898	0.0963605	5.77×10^−3^
1.0	3.2387×10^−17^	-7.702×10^−17^	1.09×10^−17^

In case of oscillation, it can be observed in Fig (9) that the boundary layer thickness is reciprocal to the perpendicular magnetic field and the fluid motion decreases one step towards the surface of the fluid. Due to oscillation of the belt, we observed that the fluid motion is maximum at the surface of the belt and minimum at the surface of the fluid. Also, it is observed that for large values of *M*, the fluid motion increases quickly as compared to small values. The influence of gravitational parameter can be seen from Fig (10) in the oscillation case. In the presence of friction force, the gravitational effect seems to be smaller near the belt and greater at the fluid surface. By increasing *m*, the speed of fluid layer increases. The effects of non-Newtonian parameters *k*_1_ and *k*_2_ on velocity profiles are shown in Figs (11 and 12). It is observed that in the presence of magnetic field, the structure of the thin layer becomes similar with those of Ekman and classical Stokes layers. It is also observed that for all frequencies, the thickness of the hydromagnetic thin layers remain bounded. The reason is that the magnetic field controls the growth of the thin layer thickness at the resonant frequency.

## Conclusion

In this paper, the approximate solutions of unsteady MHD thin film flow of an Oldroyd-B fluid through oscillating inclined belt has been obtained using OHAM and HPM methods for velocity field. Both of these solutions are compared numerically and graphically. It is found that the solution obtained by OHAM and HPM are in excellent agreement. The concluded remarks have been precised as follows:

- It is found that for a specific region *y*ϵ[0,1], thin film near the belt oscillates together with the belt in the same period and the velocity amplitude of the fluid layer increases gradually towards the free surface of the belt.
- Due to no-slip condition, the force of friction reduces the gravitational effect near the belt and this effect seems to be greater at the fluid surface.
- Since magnetic field controls the growth of thin film thickness, therefore, the thickness of thin film remains the same for different frequencies.
